# Examining the use of health systems and policy research in the health policymaking process in Israel: views of researchers

**DOI:** 10.1186/s12961-016-0139-7

**Published:** 2016-09-01

**Authors:** Moriah E. Ellen, John N. Lavis, Joshua Shemer

**Affiliations:** 1 Jerusalem College of Technology, Ha-Va’ad ha-Le’umi Street 21, Jerusalem, 93721 Israel; 2 Israeli Center for Technology Assessment in Health Care, Gertner Institute, Sheba Medical Center, Tel Hashomer, 52621 Israel; 3 McMaster Health Forum, McMaster University, 1280 Main Street West, Hamilton, ON L8S 4L6 Canada; 4 Institute for Health Policy, Management and Evaluation, University of Toronto, 4th Floor, 155 College St, Toronto, ON M5T 3M6 Canada; 5 Centre for Health Economics and Policy Analysis, McMaster University, 1280 Main Street West, Hamilton, ON L8S 4L6 Canada; 6 Department of Political Science, McMaster University, 1280 Main Street West, Hamilton, ON L8S 4L6 Canada; 7 Department of Global Health and Population, Harvard School of Public Health, 677 Huntington Ave, Boston, MA 02115 United States of America; 8 Sackler School of Medicine, Tel Aviv University, P.O. Box 39040, Tel Aviv, 6997801 Israel

**Keywords:** Health systems and policy, Knowledge transfer and exchange, Researchers, Survey

## Abstract

**Background:**

All too often, health policy and management decisions are made without making use of or consulting with the best available research evidence, which can lead to ineffective and inefficient health systems. One of the main actors that can ensure the use of evidence to inform policymaking is researchers. The objective of this study is to explore Israeli health systems and policy researchers’ views and perceptions regarding the role of health systems and policy research (HSPR) in health policymaking and the barriers and facilitators to the use of evidence in the policymaking process.

**Methods:**

A survey of researchers who have conducted HSPR in Israel was developed. The survey consisted of a demographics section and closed questions, which focused on support both within the researchers’ organisations and the broader environment for KTE activities, perceptions on the policymaking process, and the potential influencing factors on the process. The survey was sent to all health systems and policy researchers in Israel from academic institutions, hospital settings, government agencies, the four health insurance funds, and research institutes (n = 107). All responses were analyzed using descriptive statistics. For close-ended questions about level of agreement we combined together the two highest categories (agree or strongly agree) for analysis.

**Results:**

Thirty-seven respondents participated in the survey. While many respondents felt that the use of HSPR may help raise awareness on policy issues, the majority of respondents felt that the actual use of HSPR was hindered for many reasons. While facilitators do exist to support the use of research evidence in policymaking, numerous barriers hinder the process such as challenges in government/provider relations, policymakers lacking the expertise for acquiring, assessing, and applying HSPR and priorities in the health system drawing attention away from HSPR. Furthermore, it is perceived by a majority of respondents that the health insurance funds and the physician organisations exert a strong influence in the policymaking process.

**Conclusions:**

Health system and policy researchers in Israel need to be introduced to the benefits and potential advantages of evidence-informed policy in an organised and systematic way. Future research should examine the perceptions of policymakers in Israel and thus we can gain a broader perspective on where the actual issues lie.

## Background

All too often, health policy and management decisions are made without making use of or consulting with the best available research evidence [1, 2]. Decisions that are not based on the available evidence can lead to inefficient and ineffective health systems [3]. Evidence-informed policymaking, an approach that aims to ensure that decision-making is informed by the best available research evidence at the time of the decision [3], has gained traction over the last decade. Ensuring the use of research and evidence in health system management, policy- and decision-making is an important challenge in this century [4].

Initiatives and activities aimed at increasing the use of research evidence in management and decision- and policymaking have been referred to in many different ways, including knowledge transfer and exchange, knowledge translation, research utilisation, evidence-based decision-making, knowledge uptake, research implementation, research uptake, and research transfer [5–8]. For the purpose of this research, we will use the term knowledge transfer and exchange (KTE) and take it to generally encompass the previous mentioned terms. KTE has emerged as a paradigm to address many of the challenges and start closing the “know-do” gap between knowledge producers and knowledge users [1]. We define KTE as “*the synthesis, exchange, and application of knowledge by relevant stakeholders to accelerate the benefits of global and local innovation in strengthening health systems and improving people’s health*” [8]. The use of research evidence for clinical practice is well accepted and is evident in the proliferation of the development and usage of clinical practice guidelines and clinical pathways. Nevertheless, the use of research evidence in policymaking has not reached the same state of maturity. Numerous KTE frameworks and approaches have been proposed; however, a comprehensive framework still does not exist to assist in better understanding the influences on evidence-informed decision- and policymaking [4, 9–11]. It is apparent, from the frameworks and research, that the local climate and context, poor relationships between knowledge producers and users, and the perceptions of the knowledge producers regarding the roles they need to play in order to transfer the knowledge, can act as barriers to the KTE process [12–15]. The objective of this study is to explore Israeli health systems and policy researchers’ views and perceptions regarding the role of health systems and policy research in health policymaking and the barriers and facilitators to the use of evidence in the policymaking process.

Israel is a small country with a universal healthcare system, i.e. every Israeli resident is entitled to a basic package of healthcare. The main providers of the Israeli health system are the Ministry of Health, four health insurance funds and other non-profit organisations. The health system is financed through taxation linked to income, healthcare spending is approximately 8% of the GDP, and Israel has comparatively good health outcomes [16, 17]. With respect to research, health services research and biomedical research are well developed in Israel [16]. In the area of health services research, most researchers are based either at stand-alone research and policy centres, universities, the Ministry of Health, the health insurance funds, or governmental agencies, and it is common for researchers to have positions in a number of the aforementioned organisations. Health services researchers receive funding from governmental and non-governmental sources, both national and international. The main funding source comes from the National Health Insurance Law, which sets aside 0.1% of the funds collected via the health tax for research on healthcare services [16]. Researchers also at times receive government contracts to conduct specific work, depending on their area of expertise. Many researchers also partner with international teams to conduct both national and international research. Additionally, researchers in Israel try to obtain funding grants outside of Israel as they are generally for longer lengths of time and larger grants.

Among the many factors to consider, when considering evidence-informed policymaking, are the actors at play. There are two main actors in the KTE process, namely the knowledge users (i.e. decision-makers, policymakers) and the knowledge producers (i.e. researchers). Knowledge producers and users generally operate in two different communities: where one actor produces research and the other makes decisions, and rarely do the two communities cross paths [9, 18–20]. Knowledge producers make up the supply side of the research-to-policy process, and as producers of knowledge and evidence, generate the raw data needed to make crucial and far-reaching policy decisions [21]. On the opposite end, knowledge producers can also form a sounding board for policymakers. While it is tempting for policymakers’ thinking to only extend into the short-term (i.e. fixing the most immediately foreseeable problem, responding to the vociferous voter demands), researchers have other priorities, and are able to offer a different point of view [21].

Understanding the perceptions and attitudes of both knowledge users and knowledge producers on the policymaking process and the use of evidence to inform policy is important because it helps bridge the existing gaps between research, policy and practice, a process in which WHO and other stakeholders have strong interests [22]. Recently, much emphasis has been placed on policymakers, both in understanding their perceptions and in providing potential interventions [23–26]. Recent research in Israel on policymakers’ views on the role of health systems and policy research (HSPR) in the health policymaking process demonstrated that, while policymakers value the use of HSPR in the policymaking process, the actual use was hindered for many reasons, including the lack of relevant and timely research [27]. Less emphasis has been placed on understanding researchers’ perceptions on the policymaking process and the actions they take to promote evidence-informed decision-making. Of the few studies examining researchers’ KTE activities and perceptions, the outcomes are somewhat similar. Lavis et al. [28] questioned researchers from 10 low- and middle-income countries and found that less than 50% engaged in promising KTE, or ‘bridging’ activities, leaving potential areas for future initiatives to focus on supporting more bridging strategies. Some of Lavis’ findings were replicated in a 2012 study by El-Jardali et al. [29], who conducted a similar study involving 12 countries from the Eastern Mediterranean region. The majority (76%) also felt that policy formulation is based on elements other than evidence-based processes such as donor preferences and internal Ministry of Health discussions. A comparable majority also felt that there is insufficient evidence about how health policies are being made, and that there is a lack of co-ordination between stakeholders in policymaking, possibly because of their derision toward the value of evidence. It was again found that less than half of researchers engaged in knowledge transfer activities, possibly because of researchers’ reports of low support levels and lack of incentives. Researchers in Argentina [21] had similar sentiments about the lack of support and incentives, but also experienced an increased distrust for policymakers, and even other researchers, due to the socio-political history of their country.

Therefore, while researchers may have a large role to play in the evidence-informed health policy process, these studies demonstrate that researchers are minimally engaged in transferring their findings to knowledge users [30]. In Israel, a recent study demonstrated similar results in that less than half of the health systems and policy researchers were engaged in KTE activities, i.e. less than half of the health systems and policy researchers in Israel reported that they frequently or always interacted with the target audience through the research process (i.e. during developing a research question or executing the research) or through formal or informal meetings during conferences, workshops or conversations [30]. The conundrum that remains based on the results of that study is why there is limited engagement in KTE activities. Numerous explanations exist, such as the sample was not fully representative, health system and policy researchers in Israel have yet to be exposed to appropriate KTE interventions, and/or that the researchers perceive that there are other influences on the policymaking process and, therefore, maybe investing in KTE initiatives will only see limited return on investment. Understanding the views and perceptions of researchers on the policymaking process can help us better understand the phenomenon. Perceptions can influence the policymaking process and the level of KTE activities [21, 31]. Therefore, the purpose of this paper is to report the results of a study examining the views and perceptions of health systems and policy researchers in Israel on this issue.

## Methods

### Developing the survey

A cross-sectional web-based survey of health systems and policy researchers in Israel was developed. The survey was based on previous surveys and focused on support both within the researchers’ organisations and the broader environment for KTE activities, perceptions on the policymaking process, and the potential influencing factors on the process [22, 29]. The survey consisted of a demographics section and quantitative questions. The quantitative section consisted of seven main scales, all answered on a 5-point Likert scale ranging from 1 (strongly disagree) to 5 (strongly agree). In the quantitative section, the first scale consisted of four items that assessed accountability regarding KTE activities, the second scale consisted of 16 items addressing the researchers views about the barriers and facilitators for KTE, the third scale consisted of three items addressing the support for KTE within their organisation, the fourth scale consisted of five items addressing their views about the research itself and its possible influence on the policymaking process, the fifth scale consisted of three items concerning the factors that influence health policymaking in Israel, the sixth scale consisted of 11 items that explored their views on the groups or factors that could have exerted a strong influence on the health policymaking process, and the seventh scale included seven items addressing the role of HSPR and the factors that influence the use of HSPR by health policymakers and stakeholders in Israel.

The survey was web-based and no identifiers were associated with the data. The survey was piloted with one health systems and policy researcher in Israel and necessary changes were made. For example, the feedback obtained from the pilot test was that the survey was too lengthy and time consuming, and therefore the response rate would be poor. Thus, the survey focused on a limited subset of a larger pool of potential questions. The survey was also translated into Hebrew.

### Selecting the sample

Due to the size of the country and the size of this field of research in Israel, and after consulting with three senior health systems and policy professionals in Israel, it was estimated that there were approximately 60–100 health systems and policy researchers in Israel and therefore, due to the manageable size of potential respondents, it was decided that all of these would be invited to participate in the survey. The target was health systems and policy researchers from academic institutions, hospital settings, government agencies, the four health insurance funds, and research institutes (for more details on the development of the list of potential respondents, see [30]). All potential respondents were identified by (1) examining publicly available web sites associated with each of the aforementioned institution types and identifying those researchers that focused on health systems and policy, and (2) examining the list of research projects that were funded by the National Institute for Health Policy and obtaining the emails of the associated researchers (email addresses were obtained for all identified respondents). However, it is possible that some of the identified researchers did not actually focus on HSPR. Therefore, at the start of the survey, we defined health systems and policy research as “*research related to governance, financial and delivery arrangements for healthcare and population health services*” and asked respondents to only answer all subsequent questions with this type of research in mind, even if this research constitutes only a small proportion of the research with which they are involved. Thus, researchers who did not actually focus on HSPR should not have completed the survey. Finally, the snowball sampling technique was used by adding a question to the survey asking respondents to identify any additional researchers they think should be contacted that might be interested and may have relevant information for our survey; however, no new names were provided through this method.

### Survey administration

The survey was administered online using the software Survey Monkey. Survey Monkey does not require respondents to provide their names or any personal information. Respondents were approached by an email with the link to the online survey. The initial survey was in English. The survey was also administered in Hebrew through Qualtrics (due to the fact that Survey Monkey does not support Hebrew text).

We followed rigorous procedures to recruit the sample to complete the survey. An initial email was sent out, in both English and Hebrew, with a link to the survey, from the principal investigator to all potential respondents. Non-respondents were sent automated reminders from 2 weeks after the first contact and 4 weeks after the first reminder. We telephoned all potential respondents to inform them of the survey and remind them to complete the online survey (if the respondent was unavailable, and if it was possible, we left a message). Six telephone numbers were incorrect with no replacement number provided. We also employed several measures identified in a systematic review as ways to increase response rates [32]. Specifically, we personalised all emails, did not use the word ‘survey’ in the subject of the emails, shortened the questionnaire, and inserted a progress bar in the actual survey so that participants knew how far along they were in the survey. We also strived to make the content of the survey interesting to respondents and formatted the survey in a user-friendly format. Most importantly, in the systematic review, it was found that the odds of response were increased by more than a half using non-monetary incentives, and therefore we offered the participants a chance to win an iPad. After completing the survey, participants were redirected to a separate website where they could enter their name and contact information and were entered into a draw to win an iPad.

### Data analysis

All the quantitative responses were exported from the web-based surveys to the Statistical Package for Social Sciences (SPSS) for analysis and analyzed using descriptive statistics. For close-ended questions about level of agreement we combined together the two highest categories (agree or strongly agree) for analysis. Mann–Whitney non-parametric tests were carried out to examine the differences by gender, Spearman correlations to examine differences by age, and Kruskal–Wallis nonparametric tests were carried out in order to examine differences by education and affiliation. We analyzed the responses based on different demographic variables as we wanted to see if there was a difference in responses. We also analyzed the responses by affiliations because different organisations can have different experiences with HSPR and different interactions with decision-makers and we wanted to explore if respondents with different affiliations had different views and experiences.

## Results

Overall, 107 health system and policy researchers were invited to participate in the survey: 37 responded, for a response rate of 35%. Not all the respondents fully completed the survey; however, because we analyzed the data descriptively, per item, we decided not to remove their responses from the analysis.

### Demographics

Among the 37 respondents, 16 were males and 17 females. The average age of the respondents was 51.9 (SD 10.9) years. The age range was 30 to 68 years. Sixteen respondents (44.4%) were affiliated with an academic university, 12 (33.3%) were affiliated with a research institute not within a university and 7 respondents (19.4%) work in a teaching hospital setting. Only one respondent was affiliated with a government department or agency. The majority of respondents (62.2%) had a doctorate degree.

### Perceptions of the role of HSPR in influencing the health policymaking process

While 54% of health systems and policy researchers in Israel perceive that evidence from health systems and policy research does help health policymakers and stakeholders to identify and/or choose policy alternatives, the majority of respondents felt that the actual use of evidence from health systems and policy research was hindered for various reasons, i.e. practical constraints (68%) and lack of coordination between policymakers and researchers (59 %) (Table 1). Close to half of the respondents perceived that the use of health systems and policy research was hindered by a non-receptive policy environment or by findings that were politically insensitive or inconsistent with a policy direction.

**Table 1:** The role of health systems and policy research (HSPR) and the factors that influence the use of HSPR by health policymakers and stakeholders in Israel

	Percentage agree or strongly agree (n = 34)
Use of evidence from HSPR in policy was hindered by practical constraints to implementation such as financial implications	68
Evidence from HSPR does help raise health policymakers and stakeholders’ awareness on policy issues^a^	65
Lack of coordination between policymakers and researchers hindered the use of evidence from HSPR in the health policymaking process	59
Evidence from HSPR does help health policymakers and stakeholders to identify and/or choose policy alternatives^a^	54
Use of evidence from HSPR in policy was hindered by a non-receptive policy environment	47
Use of evidence from HSPR in policy was hindered by findings that were politically sensitive or were inconsistent with a policy direction	47
Evidence from HSPR was presented to policymakers and stakeholders in a timely manner and in a format that they can understand	34

^a^The N for these two questions was 35

### Perceptions of barriers and facilitators for KTE

With respect to activities that facilitate KTE, more than half of the health system and policy researchers felt that the national funding organisations formulated their funding calls in response to regional and national needs (Table 2). Approximately 40% felt that contacts, both personal and organisational with policymakers were stable over time, that funding sources both encouraged and permitted KTE as an allowable expense, and that they had access to technical support to acquire, assess and apply the necessary research. However, more than half of respondents felt that policymakers do not make decisions based on health systems and policy research. Numerous barriers were identified that could explain why. For example, more than half of the health system and policy researchers felt that policymakers did not have the necessary skills for acquiring, assessing and applying the relevant research and that they do not make decisions based on health systems and policy research. Furthermore, close to 60% of the respondents felt that other priorities in the health system drew attention away from health systems and policy research. Less than a quarter of health system and policy researchers felt that organisations that conduct health systems and policy research assisted with KTE activities by making financial and human resources available to assist in the transfer of knowledge (Table 3). A little over half of the researchers feel that the currently available research aligns with the needs of the knowledge users and less than half felt that the currently available research aligned with the country’s priorities (Table 4).

**Table 2:** Potential facilitators and barriers to the use and implementation of knowledge translation and exchange (KTE) activities

Factors	Percentage agree or strongly agree (n = 37)
Facilitators:	
National funders formulate their priorities and calls for proposals in response to national and regional needs	59
Personal and organisational contacts among policymakers were quite stable over time	43
Funding sources (e.g. granting agencies) encourage engagement in KTE activities	43
Funding sources (e.g. granting agencies) consider KTE activities an allowable expense	43
Policymakers have access to technical support for acquiring, assessing and applying health systems and policy research (HSPR) research	42
Structures and processes exist to link you with policymakers	38
National funding sources encourage KTE activities	38
Policymakers invest financial and/or human resources in KTE activities	22
Policymakers create opportunities to develop joint HSPR research initiatives with them	22
Barriers:	
Policymakers lack the expertise for acquiring, assessing and applying HSPR research	59
Priorities in the health system draw attention away from HSPR research	59
Policymakers do not make decisions on the basis of HSPR research	53
Policymakers and stakeholders consider that the available HSPR has little practical policy applications	38
Policymakers do not have technical access (i.e. journal subscriptions, links to research) to the appropriate databases to search for HSPR research	32
Policymakers and stakeholders consider that the available HSPR lacks credibility	24

**Table 3:** Additional facilitators and barriers at the level of organisational support for knowledge translation and exchange (KTE) activities

	Percentage agree or strongly agree (n = 37)
Knowledge translation was hampered by a lack of incentives for KTE activities within organisations that conduct health systems and policy research (HSPR)	38
Organisations that conduct HSPR made available financial and human resources to assist with KTE activities	24
Organisations that conduct HSPR were not seen as a credible source of research	14

**Table 4:** Alignment of available research to needs of knowledge users

	Percentage agree or strongly agree (n = 37)
Available research coincided with the needs and expectations of target audiences	51
Available research coincided with my country’s priorities (e.g. with a National Research Agenda)	43
Available research was not considered relevant by policymakers	28
Available research lacked credibility among target audiences	14
No research was ready for use	5

### Perceptions of who should be responsible for KTE activities and what influences the health policymaking process in Israel

An overwhelming majority felt that KTE activities were a collective responsibility between knowledge producers, i.e. researchers, and knowledge users, i.e. policymakers (Table 5). The majority of health systems and policy researchers in Israel felt that challenges in intergovernmental relations between ministries and government/provider relations were a hindrance to the policymaking process (Table 6). Furthermore, the perception among the majority of the respondents (62%) was that the policymaking process was not usually based on evidence-based processes. The three main influencing factors that were perceived to have exerted a strong influence on the health policymaking process were health insurance funds (92%), physician associations (89%) and limited health funding (88%) (Table 7). More than half of the respondents also felt that the media (70%), values of the governing parties (61%) and public opinion (53%) were a strong influence on the health policymaking process.

**Table 5:** Views about who should be responsible for knowledge translation and exchange (KTE) activities

	Percentage agree or strongly agree (n = 37)
Research organisations, researchers, policymakers and stakeholders are jointly responsible for KTE activities	84
Research organisations are primarily responsible for KTE activities	70
Policymakers and stakeholders are primarily responsible for KTE activities	51
Researchers who conduct research on the health topic are primarily responsible for KTE activities	43

**Table 6:** Factors that influence health policymaking in Israel

	Percentage agree or strongly agree (n = 37)
Broad challenges in intergovernmental (i.e. Ministry of health, Ministry of Finance) relations hindered the health policymaking process	76
Broad challenges in government/provider relations hindered the health policymaking process	70
Policy formulation is usually based on internal Ministry of Health discussions and ad hoc process rather than evidence-based processes	62

**Table 7:** Groups or Factors that exert a strong influence on the health policymaking process

	Percentage agree or strongly agree (n = 36)
Health insurance funds	92
Physician associations	89
Limited health funding (the economy)	88
Media	70
Values of governing parties	61
Public opinion	53
Nursing associations	46
Research about problems related to healthcare or health systems	39
Other countries’ health policies	30
Donor organisations	22
Other types of health professional associations	22

### Differences in responses based on researchers’ affiliation and gender

The difference in responses based on different demographic groups was examined. Mann– Whitney nonparametric tests were carried out in order to examine the differences by gender in the research questions. The only significant difference was found in the question: “Research organisations, researchers, policymakers, and stakeholders are jointly responsible for KTE activities” (Z = –2.173, *P* < 0.05). Females tended to strongly agree with the statement significantly more the males. Kruskal–Wallis nonparametric tests were carried out in order to examine the differences by researchers’ affiliation. The items in which significant differences were found are presented in Table 8. In many of the items presented, the respondents from the research institutes responded at the other end of the spectrum, as opposed to the respondents from the academic universities and the teaching hospitals that were clustered relatively close together.

**Table 8:** Difference in responses based on researchers’ primary affiliation

	Academic university n = 14		Teaching hospital setting n = 7		Research institute n = 12		Kruskal–Wallis χ^2^(2)
	Mean	Standard deviation	Mean	Standard deviation	Mean	Standard deviation	
Policymakers invest financial and/or human resources in joint HSPR research initiatives with them	2.31	0.95	2.14	1.21	3.25	0.97	6.770*
Knowledge translation was hampered by a lack of incentives for knowledge translation activities within organisations that conduct HSPR	3.56	0.96	3.57	0.53	2.67	0.89	6.755*
Organisations that conduct HSPR were not seen as a credible source of research	2.69	1.14	2.71	1.11	1.58	0.67	9.038*
Policy formulation is usually based on internal Ministry of Health discussions and ad hoc process rather than evidence-based processes	3.94	0.85	3.00	0.82	3.33	0.98	6.649*
Broad challenges in intergovernmental (i.e. Ministry of Health, Ministry of Finance) relations hindered the health policymaking process	4.47	0.64	3.71	0.95	3.46	0.88	9.669**
Broad challenges in government/provider relations hindered the health policymaking process	4.20	0.68	3.57	0.79	3.08	1.00	9.643**
Values of governing parties (i.e. groups or factors exerted a strong influence on the health policymaking process)	4.40	0.63	3.43	0.79	3.42	1.00	9.597**

*P < 0.05; **P < 0.01

## Discussion

### Summary of study findings

The main purpose of this study was to examine Israeli health systems and policy researchers’ views and perceptions regarding the role of health systems and policy research in health policymaking and the barriers and facilitators to the use of evidence in the policymaking process. While many respondents felt that the use of HSPR may help raise awareness on policy issues, the majority of respondents felt that the actual use of HSPR was hindered for many reasons. The findings suggest that the researchers view the use of evidence in decision-making as a mutual responsibility, and while facilitators do exist to support the use of research evidence in policymaking, numerous barriers hinder the process. There are strong overarching barriers to the policymaking process in general, such as challenges in government/provider relations, but also specifically to the use of research, such as policymakers lacking the expertise for acquiring, assessing and applying health systems and policy research and priorities in the health system, thus drawing attention away from HSPR. Furthermore, it is perceived by a majority of respondents that the health insurance funds and the physician organisations exert a strong influence in the policymaking process.

The difference in responses between organisation types could be attributed to a number of reasons. The respondents were asked to identify their primary affiliation and were given three main choices: academic universities, teaching hospitals or research institutes. Researchers that responded from research institutes were affiliated with one of two research institutes, both focusing on health policy research in Israel. Researchers within these institutes typically work closely with policymakers or are called upon by policymakers or decision-makers to conduct specific research studies or provide information related to a pertinent decision. Due to their close affiliation and association with policymakers, it is conceivable that respondents from these types of organisations responded more favourably to statements that reflected positively on policymakers, such as “*Policymakers invest financial and/or human resources in joint HSPR research initiative*”; or more negatively to statements that portrayed policymakers or the policymaking process in a negative light, such as “*Broad challenges in government/provider relations hindered the health policymaking process*”. The researchers that work at the research institutes are much closer to the policymaking process so therefore their perceptions may be quite different than those that have their primary affiliation in an academic university or a teaching hospital.

### Relation to other studies

The findings in this study align with recent studies about KTE that used similar tools and found that health systems and policy researchers perceived there to be numerous barriers and minimal support to the use of evidence in policymaking [22, 29]. Similar to this study, other studies found that researchers may feel that their work is not utilised because of competing interests, lack of funding, or a lack of will on the decision-makers’ part [29]. In Lavis’ 2010 study [22], over a third of researchers did not feel that their country’s health research environment was supportive of individuals undertaking knowledge translation activities (37%) and nearly half (47%) did not feel that there were sufficient structures and processes in place to link researchers and their target audience. A qualitative study that was conducted in Argentina also demonstrated that researchers’ perceived that “*policymakers were unlikely to use evidence when developing policies*” and they identified numerous barriers to the use of evidence to inform policy such as political governance, the bureaucratic process and an overall lack of trust within the system [21]. In comparing the results of the present study to previous ones, a main difference between the findings was identified in relation to the groups or factors that exert a strong influence on the health policymaking process (Table 6). Herein, it was identified that health insurance funds and physician organisations exerted considerable influence over the policymaking process, whereas in El Jardali et al.’s [29] study only 37%, 27% and 29% felt that physician associations, private insurers and public health providers, respectively, exerted a strong influence in the health policymaking process. The difference in these outcomes is possibly due to the way that the health system is structured in Israel as opposed to in the rest of the Eastern Mediterranean countries (the focus of El-Jardali’s study) [29]. In Israel, since 1995 when the National Health Insurance Law came into effect, membership in one of the four existing health insurance funds is compulsory for all Israeli citizens. Therefore, the four health insurance funds are strong players in the system and their interests can strongly influence health policy discussions.

Interests, institutions, ideas and external forces play a strong role in the policymaking process [33, 34], and while KTE researchers strive for evidence-based policymaking, in reality, the attainment of evidence-informed policymaking is somewhat more realistic. Evidence only plays one role (and unfortunately it is sometimes a small one) in the policymaking process and external factors, political and institutional forces usually have a stronger role in the policymaking process. Numerous studies have identified various external forces that influence the policy process such as external donors, trade unions, organisations or the pharmaceutical industry [31, 35–37]. These external groups and influences have the potential to act as either barriers or facilitators, depending on the issue at hand and the management of the process [31, 37]. Based on this study, the strongest influencing groups are the health insurance funds and the physician organisations, and further interventions should consider engaging these stakeholders to help facilitate the implementation of an evidence-informed policymaking process. According to previous research conducted in Israel, less than half of the health systems and policy researchers are engaged in KTE activities [30]. Considering these strong influences, it may be worthwhile to collaborate from the beginning with the health insurance funds and physician organisations on KTE initiatives to try and ensure successful implementation.

While researchers are an indispensable group that needs to be considered when discussing evidence-informed policymaking, there has been minimal work done examining perceptions of researchers. However, much more work has attempted to discern decision-makers’ perceptions on the knowledge transfer process. For example, a systematic review of the literature found that one of the prevailing barriers to the use of research in health system decision-making, as well as a root cause for gaps between research, policymaking and implementation, is decision-makers’ perception of health systems and policy research and researchers, as well as their role in the policymaking process [31]. In Israel, a study on policymakers’ views on the role of HSPR demonstrated that, while there are many barriers, there are numerous facilitators that are already in place and support evidence-informed policymaking such as the strong relationships and collaborations between knowledge users and knowledge producers [27]. However, knowledge users identified areas for improvement within these collaborations, e.g. partnering in designing and executing research projects to ensure their relevance. The health system policymakers in Israel were open to receiving relevant research in effective formats and using research in decision-making, yet, assisting knowledge users in acquiring and assessing the relevant knowledge is not done often enough [27]. This current study is only a small step toward an improved knowledge of the views of researchers – vital stakeholders in the health decision-making process, who produce the evidence on which health policy decisions must be based.

### Strengths and limitations

Our study has three main strengths: (1) the survey itself was built on a pre-existing and validated instrument, (2) numerous initiatives were undertaken to ensure a high response rate (i.e. the survey was computerised and web-based, it was disseminated in both Hebrew and English, and we used non-monetary incentives to try and increase the response rate), and (3) it is the first study to explore the perceptions of health systems and policy researchers on KTE and the health policymaking process in Israel. However, our survey is not without limitations. The main limitation is that the survey is based on self-reports and, therefore, the possibility that the responses may be biased due to social desirability bias cannot be excluded. Furthermore, due to the nature of the self-selection of the sample, one could argue that only those researchers with exposure to KTE chose to participate in the study and therefore the results do not properly reflect what occurs in actuality. An additional limitation is that, although we took numerous steps to develop as thorough a list as possible of potential respondents, it is possible that there are some researchers that were not identified, thus limiting the representativeness of the sample. A further limitation is that, despite our efforts to increase response rates, the actual response rate was relatively low, which affects the generalisability of the findings. The low response rate, and therefore the small sample size of the responses analysed, can have two effects that may influence analysing and understanding the results. One issue is the uncertainty surrounding the size of the point estimate – the percentage of respondents agreeing or strongly agreeing with each statement. In this case, the confidence interval was relatively wide. It is interesting to note, however, that, despite the significant uncertainty, for questions on which a large majority of the respondents agreed, i.e. “*Research organisations, researchers, policymakers, and stakeholders are jointly responsible for KTE activities*” and that health insurance funds, physician associations and the economy all have a strong influence on the policymaking process, the lower bound of the confidence interval was 70% and higher, which underlines the fact that it is indeed a majority opinion. Similarly, the upper bound of the confidence interval on questions where most respondents disagreed (e.g. policymakers invest resources in KTE activities, policymakers consider the available HSPR as lacking in credibility or that no research was ready for use) was well below 50%, indicating that those who agreed with those statements were a minority.

The other issue is that the small sample size limits the ability to detect differences within groups. The fact that significant differences were found for eight questions even with a sample of 14 respondents from academic universities, seven from teaching hospitals and 12 from research institutes, is a testament to the degree of difference in experience and perspective between them. It is possible that, with a larger sample, other differences would have become more apparent.

### Implications for policy and practice and future research

The use of research evidence in health policymaking is not as self-evident as it is tempting to assume. Barriers for the lack of use of research in policymaking have been identified in numerous papers and activities that can be undertaken by researchers and knowledge users have been identified as a way of bridging the divide [31, 38, 39]. Researchers and policymakers are starting to engage in efforts to collaborate and implement linkage and exchange opportunities due to global calls for promoting the application of research evidence in policymaking [40, 41]. Understanding the perceptions of both parties, i.e. researchers and knowledge users, on the health policymaking process and the use of research in policymaking within the Israeli context is imperative as there has been little previous work done in the area and knowledge is limited [30]. A recent study examining KTE activity in Israel found a lack of initiatives in the field [30]. This could be because the idea of KTE is relatively novel to the country; however, there is considerable promise in spreading the idea, as Israel is a small place where the community of researchers and users is more tightly knit [27, 30]. This study provides insight as to the perceptions of researchers on the policymaking process and the different factors that influence the process. Future research should examine the similarities and differences in perceptions of policymakers and researchers in Israel and thus we can gain a broader perspective on where the actual issues lie. Furthermore, qualitative interviews with researchers could explain why limited KTE initiatives have been pursued, how the perceptions have influenced their activities related to evidence-informed policy and future interventions that should be explored. Building on this study and previous work, it is time to start developing, implementing and evaluating interventions to assist in the dissemination of timely and efficient research to policymakers. Considering the results of this study, this should be done in partnership and with the buy-in of policymakers and the various actors with strong interests in the policymaking process in order to ensure success.

## Conclusions and future research

This research demonstrated that just over half of the health systems and policy researchers in Israel perceive that evidence from health systems and policy research does help health policymakers and stakeholders to identify and/or choose policy alternatives; however, the majority of respondents felt that the actual use of evidence from health systems and policy research was hindered for several reasons and a variety of barriers. KTE is a fairly new area in Israel and therefore many barriers to the use of HSPR in the policymaking process exist. This study is part of a larger proposed program of research that demonstrates the minimal engagement that health systems and policy researchers in Israel have undertaken within a growing field of KTE. Health systems and policy researchers in Israel need to be introduced to the benefits and potential advantages of KTE in an organised and systematic way, yet, based on this research, this must be done in a collaborative way with the various health system stakeholders.

## Abbreviations

HSPR: Health systems and policy research
KTE: Knowledge transfer and exchange
